# Identifying Rhodamine Dye Plume Sources in Near-Shore Oceanic Environments by Integration of Chemical and Visual Sensors

**DOI:** 10.3390/s130303776

**Published:** 2013-03-18

**Authors:** Yu Tian, Xiaodong Kang, Yunyi Li, Wei Li, Aiqun Zhang, Jiangchen Yu, Yiping Li

**Affiliations:** 1 State Key Laboratory of Robotics, Shenyang Institute of Automation, Chinese Academy of Sciences, Shenyang 110016, China; E-Mails: tiany@sia.cn (Y.T.); kangxdcsu@163.com (X.K.); zaq@sia.cn (A.Z.); yjc@sia.cn (J.Y.); lyp@sia.cn (Y.L.); 2 Department of Computer & Electrical Engineering and Computer Science, California State University, Bakersfield, CA 93311, USA; E-Mail: yl180@duke.edu

**Keywords:** insect-inspired robots, chemical plume tracing, autonomous underwater vehicle, odor source identification, fuzzy color segmentation

## Abstract

This article presents a strategy for identifying the source location of a chemical plume in near-shore oceanic environments where the plume is developed under the influence of turbulence, tides and waves. This strategy includes two modules: source declaration (or identification) and source verification embedded in a subsumption architecture. Algorithms for source identification are derived from the moth-inspired plume tracing strategies based on a chemical sensor. The in-water test missions, conducted in November 2002 at San Clemente Island (California, USA) in June 2003 in Duck (North Carolina, USA) and in October 2010 at Dalian Bay (China), successfully identified the source locations after autonomous underwater vehicles tracked the rhodamine dye plumes with a significant meander over 100 meters. The objective of the verification module is to verify the declared plume source using a visual sensor. Because images taken in near shore oceanic environments are very vague and colors in the images are not well-defined, we adopt a fuzzy color extractor to segment the color components and recognize the chemical plume and its source by measuring color similarity. The source verification module is tested by images taken during the CPT missions.

## 1. Introduction

Autonomous underwater vehicles (AUVs) with chemical plume tracing (CPT) capabilities would be valuable in searching for deep sea hydrothermal vents, finding unexploded ordnance in near-shore oceanic environments, and monitoring pollutants or localizing sources of hazardous chemicals in a harbor. Factors that complicate CPT in natural environments (near-shore ocean conditions) include significant filament intermittency and chemical plume meander, and flow variations with both location and time.

Over the last decade there has been interest in developing bio-inspired strategies for CPT in natural environments. Belanger and Willis presented plume tracing strategies, including counter-turning strategies, intended to mimic moth behavior and analyzed the performance in a computer simulation [1]. Li *et al.* evaluated and optimized the moth-inspired plume tracing strategies using a simulated plume with significant meander and intermittency of plume puffs [2]. Grasso and Basil presented biomimetic strategies, inspired by lobsters, crayfishes, and crab, and used a lobster-inspired robot for locating an odor source [3]. Minagawa *et al.* presented a crayfish robot employing flow induced by waving to locate a chemical source [4]. Lochmatter and Martinoli tested the bio-inspired algorithms for tracking odor plumes in a laminar wind field [5]. Swarm-based CPT algorithms inspired by insects, such as a group of moths or an ant colony, are discussed in [6–8]. Most engineering-oriented algorithms for CPT are comprehensively reviewed in [9].

Most of the existing studies have mainly focused on the plume tracing issue and validated their algorithms in a small operation area in a range of centimeters to a few meters or in a simulated environment, but they lacked detailed reports of CPT experiments in natural environments on a large scale. The tracking of a chemical plume in the natural environment, such as near-shore oceanic conditions, where the plume is developed under the influence of turbulence, tides and waves, challenges CPT algorithms on how to cope with the intermittency of filaments and significant plume meander. The lack of correlation between the fluid flow direction and the plume long axis often causes an AUV to lose contact with the plume because the instantaneous fluid flow direction within a plume with significant meander is not always aligned with the plume's long axis [10]. Conducting CPT missions in near-shore ocean environments on a large scale has to consider the full spectrum of field behavior that include plume finding, plume following, maneuvers to recontact a lost plume, and declaration that the source has been found [2]. Li *et al.* proposed a subsumption architecture for CPT missions [11], consisting of these four fundamental behaviors: finding the plume, maintaining the plume, reacquiring the plume, and declaring the source location or identifying the source location.

**Figure 1.**
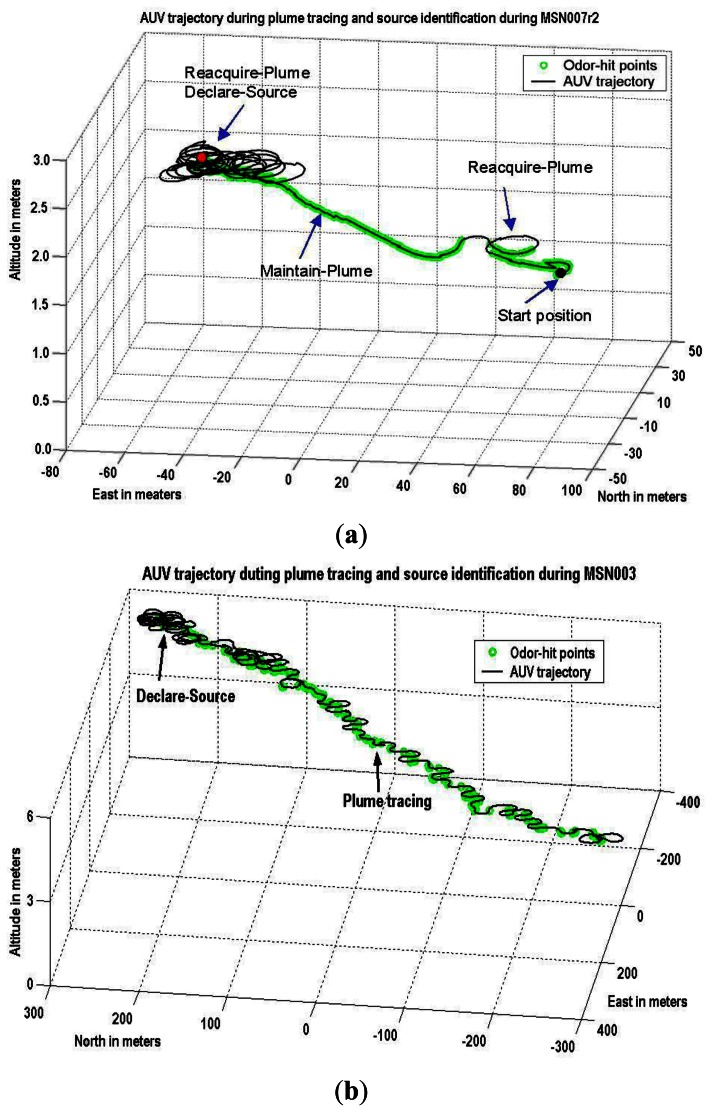
Identify the chemical source using the SIZ_T algorithm. (**a**) The first successful CPT in a near-shore ocean environment was conducted in November 2002 on San Clemente Island (California, USA). The mission MSN007r2 documented over distance of 411 meters from the first detection point to the identified source location. (**b**) The MSN003 mission at Duck (North Carolina, USA) in June 2003 tracked a chemical plume with the longest distance over 975 meters between the first point of chemical detection and the identified source location.

**Figure 2.**
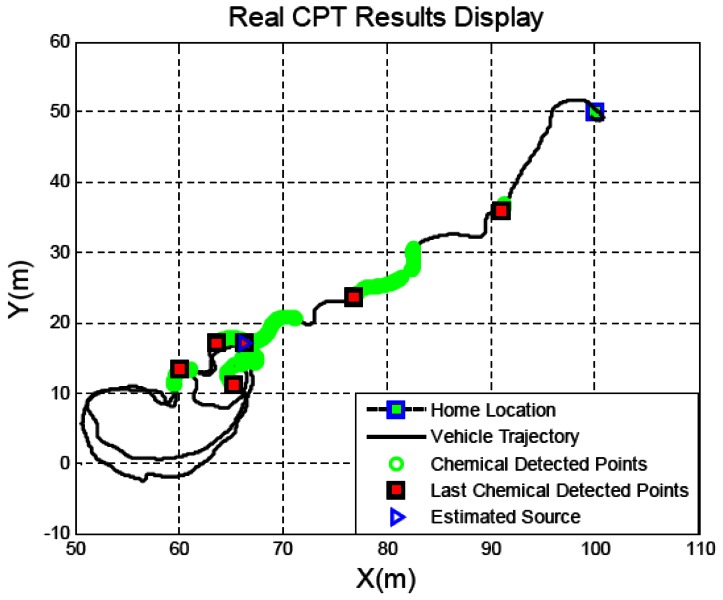
Identify the chemical source using the SIZ_F algorithm. The mission MSN171639 Dalai Bay (China) in October 2010 traveled over 60 meters from the home location to identify the source location.

Our research mainly focuses on the plume source identification issue in real world scenarios. The strategy discussed herein includes two modules: Source declaration (or identification) and source verification. This article presents the experimental results on identifying rhodamine dye plume sources in near-shore oceanic environments to validate two variations of the source identification algorithms SIZ_T and SIZ_F described in [12]. For the in-water tests, the SIZ_T algorithm was implemented on a REMUS vehicle, developed by the Oceanographic Systems Laboratory at the Woods Hole Oceanographic Institute [11,13], as shown in Figure 1. In order to validate the SIZ_F algorithm [14], Shenyang Institute of Automation (SIA), Chinese Academy of Sciences, developed a new AUV plume tracer and an alternative strategy for generating a rhodamine dye plume in near-ocean environment. Figure 2 shows the mission MSN171639 conducted at Dalai Bay (China) in October 2010.

In the field tests, several strategies were used to verify the declared chemical source, e.g., for the experiments in 2002, divers observed the declaration activities with help of an additional underwater camera system, for the experiments of 2003, side scanners generated sonar images to verify the declared chemical source, and for the experiments of 2010, an operator visually verified the declared chemical source. For further operations to deal with the declared plume source, the source needs to be automatically or interactively confirmed or inspected using a visual system. However, images taken in near shore ocean environments are very vague and colors in the images are not well-defined since the propagation of light underwater is affected by scattering, refraction, and absorption. In order to deal with these uncertainties, we adopt a fuzzy color extractor to segment the color components and recognize the chemical plume and its source by measuring color similarity.

This paper is organised as follows: In Section 2, we present the subsumption architecture for chemical plume tracing. In Section 3, we discuss the source declaration module derived from a moth-inspired plume tracing strategy. In Section 4, we report the in-water tests for declaring rhodamine dye plume source locations, mainly focusing on the most recent tests conducted in October 2012. In Section 5, we discuss the fuzzy color segmentation based verification module. In Section 6, we draw conclusions.

## 2. Subsumption Architecture for Plume Tracing

**Figure 3.**
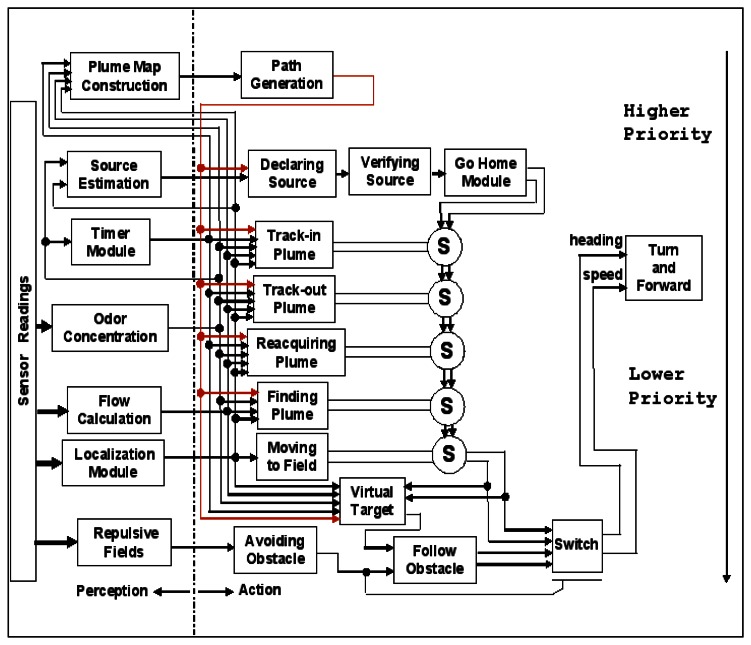
Subsumption architecture for chemical plume tracing.

A subsumption architecture [15] provides a bottom-up method of implementing a behavior-based control system layer-by-layer, so the architecture is suited to integrate the low-level bio-inspired algorithms with high-level machine intelligence algorithms. For example, the subsumption architecture for the CPT mission in Figure 3 has easily expanded from having four behaviors in [11] to having six behaviors in [16]: Finding The Plume (Find-Plume), maintaining the plume (Maintain-Plume), reacquiring the plume (Reacquire-Plume), identifying the source location (Declare-Source), avoiding an obstacle (Avoid-Obstacle), and constructing a plume map (Construct-Plume-Map). The architecture uses inhibition or suppression mechanisms to coordinate potential conflicts of the commands from the various behaviors. Consequently, the outputs from a higher layer override or subsume the output generated by behaviors in the next lower layer. Maintain-Plume and Reacquire-Plume are abstracted by the methods of location of pheromone-emitting females by flying male moths [17,18]. Find-Plume is abstracted from fluid mechanics forces [19,20]. Construct-Plume-Map and Avoid-Obstacle are discussed in [16] and [21], respectively. Different plume tracing tests in a laboratory, for CPT missions in near-shore ocean environments the go-home module controls the vehicle to go back the home location where the vehicle is launched for pick-up, after the chemical source is identified.

The objective of the source identification is to declare the chemical source location during a CPT mission. For biological entities (e.g., moths), the conclusion of identifying the pheromone source may still be a mystery. Instead, while moth plume tracing relies primarily on a sensed pheromone, the final determination on the location of the female moth could be based on data from multiple sensors, including vision, tactile, or even auditory cues [22]. This article discusses how to implement the source declaration and source verification modules embedded in the subsumption architecture. The source declaration module uses plume events detected by a chemical sensor, in combination with measured AUV locations and fluid flow directions, to identify the plume source location, while the source verification module uses fuzzy color segmentation algorithms to recognize the chemical plume and its source in an image taken when the source is declared.

## 3. Source Declaration Module

**Figure 4.**
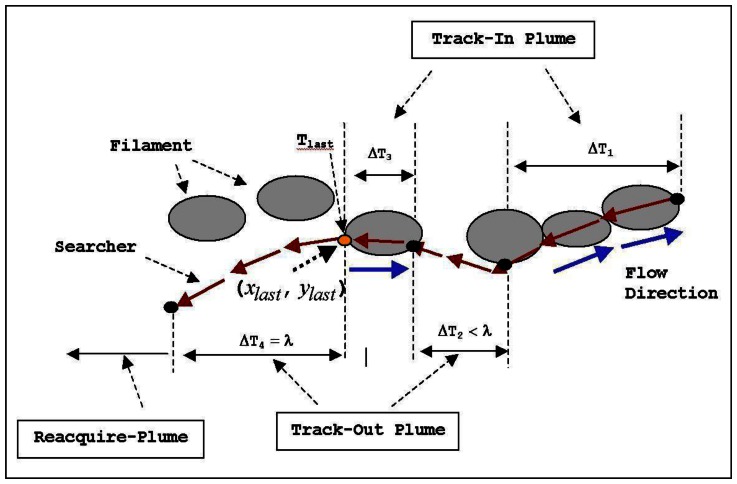

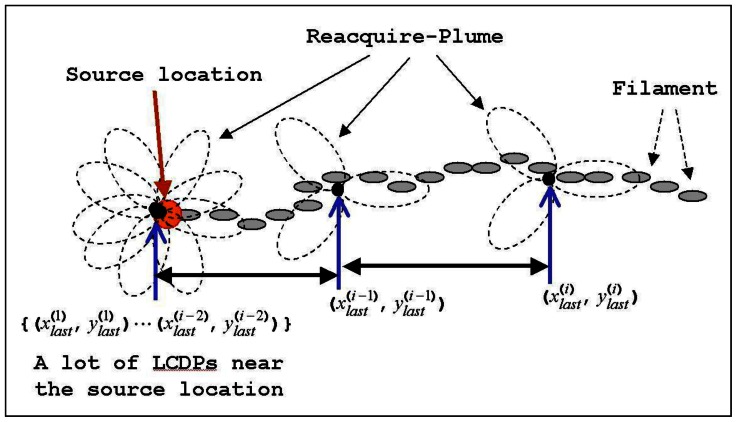
Derive source identification algorithms from moth-inspired plume tracing strategies. (**a**) An AUV records (*x_last_*, *y_last_*) as a last chemical detection point (LCDP), if it cannot re-catch the plume within λ seconds during Track-Out activity. (**b**) The AUV generates most of the LCDPs in the vicinity of the source location, when it overshoots the source and reacquires the lost plume.

This section briefly presents the source identification algorithms derived from the two moth-inspired behaviors: Maintain-Plume and Reacquire-Plume. Maintain-Plume is broken down into Track-In and Track-Out activities due to intermittency of a chemical plume transported in a fluid flow environment [12]. An AUV alternatively utilizes Maintain-Plume and Reacquire-Plume in making progress towards the source location in the up-flow direction. In a typical scenario of plume tracing, the AUV activates Track-In once it detects the chemical, e.g., the activities during Δ*T*_1_ and Δ*T*_3_ in Figure 4(a). It continues Track-Out when it loses contact with the chemical within λ seconds, e.g., the activity during Δ*T*_2_ in Figure 4(a). After λ seconds, it switches to Reacquire-Plume for casting the plume, e.g., the activity after Δ*T*_4_ in Figure 4(a).

A chemical detection point at which the AUV loses contact with the chemical plume for λ seconds is defined as a LCDP, e.g., point (*x_last_*, *y_last_*) at *T_last_* in Figure 4(a). In our applications, the coordinates of (*x_last_*, *y_last_*) are specified in a coordinate system with the origin defined by the center of an operation area. We use a Cloverleaf trajectory with the center (*x_last_*, *y_last_*) to implement the Reacquire-Plume behavior, as shown in Figure 4(b). Note that one leaf is aligned with the down-flow direction for the AUV to rediscover the chemical when it has passed the source location. During a Reacquire-Plume activity, the AUV either detects the chemical or completes the Cloverleaf trajectory *N*_re_ times (*N*_re_ = 2 or 3 for the in-waters). The length of each leaf of the Cloverleaf trajectory is constrained to be larger than the AUV turning radius about 15 meters. If *N*_re_ repetitions are completed without a chemical detection, the AUV reverts to Find-Plume. Here, we define a LCDP node by:
**struct LCDP_Node**{ **double***T_last_*, *x_last_*, *y_last_*; **double***conc*, *f_dir_*, *f_mag_*; **double***x_flow_*, *y_flow_*};where *T_last_* is the time when the LCDP is detected, (*x_last_*, *y_last_*) are the coordinates of the AUV at *T_last_*, *conc* is the chemical concentration at (*x_last_*, *y_last_*) and *T_last_*, (*f_dir_*,*f_mag_*) are the flow direction and magnitude at (*x_last_*, *y_last_*) and *T_last_*, and (*x_flow_*, *y_flow_*) are the coordinates in a new coordinate system defined according to the current flow direction. For convenience, we also use (*x_last_*, *y_last_*) to represent the LCDP in the following discussion.

In the moth-inspired CPT strategies, the chemical sensor works as a “binary detector”. The Boolean value is “1” if the chemical concentration is above the threshold. Otherwise, the Boolean value is set to “0”. The threshold value was chosen as *conc* > 4% of the full scale (*i.e.*, 0.2 V) based on an analysis of chemical sensor data from the REMUS operating in San Diego Bay in the absence of the chemical. In this scenario, the sensor readings were pure noise, but never surpassed 0.2 V. The proposed algorithms make the source identification decision based on the number of LCDPs in SIZ instead of their concentrations. LCDPs provide important information about plume traversal distances between Reacquire-Plume activities. The LCDPs are separated along the axis of the plume when the AUV is far from the source location, while the LCDPs are clustered in the vicinity of the source when the AUV is approaching the source location. The AUV usually exits the plume and moves up flow from the source when it traces the plume to the source location. When this situation occurs, the AUV also activates Reacquire-Plume to rediscover the plume on a Cloverleaf trajectory. As a result of the frequent switching between Maintain-Plume and Reacquire-Plume, the AUV generates a pattern with a number of Cloverleaf trajectories in the vicinity of the source location, as shown in Figure 4(b). Such a distribution of the LCDPs is employed to facilitate development of the source identification algorithm.

The AUV detects a new LCDP and inserts its node into the priority queue when it switches its behaviors from Maintain-Plume to Reacquire-Plume. The queue sorts the LCDP nodes in a new coordinate system, defined in order of the current up-flow direction, *f_dir_* + 180 °. Its *x* axis is aligned with the *f_dir_* direction, and its origin is located at (*x_last_*, *y_last_*). The algorithm maps each LCDP in the queue into the new coordinate system by:

(1) $$\left[\begin{array}{c} x_{\text{flow}}\\ y_{\text{flow}} \end{array}\right]=\left[\begin{array}{cc} \cos(f_{\text{dir}}+180{}^{\circ}) & -\sin(f_{\text{dir}}+180{}^{\circ})\\ \sin(f_{\text{dir}}+180{}^{\circ}) & +\cos(f_{\text{dir}}+180{}^{\circ}) \end{array}\right]\;\left[\begin{array}{c} x_{\text{last}}\\ y_{\text{last}} \end{array}\right]$$

The *x_flow_* components determine the LCDP nodes’ priorities according to the current up-flow direction. The smallest *x_flow_* has the highest priority. The more LCDPs the priority queue accumulates, the more information about the source location the AUV gathers. We use LCDPs to develop the source identification algorithms SIZ_T and SIZ_F.

Algorithm 1 lists the pseudo code of the SIZ_T algorithm which keeps updating the *N_dec_* most recent LCDPs during CPT missions, and sorts them in the order of time serials using the priority queue. The algorithm constructs the SIZ_T size by:

(2) $$\begin{array}{cc} \begin{array}{cc} x_{\text{last}}^{\left(\min\right)} & =\min\left\{x_{\text{last}}^{\left(t(\text{i}\right)}\right\}\\ x_{\text{last}}^{\left(\max\right)} & =\max\left\{x_{\text{last}}^{t(\text{i})}\right\}\\ y_{\text{last}}^{\left(\min\right)} & =\min\left\{y_{\text{last}}^{t(\text{i})}\right\}\\ y_{\text{last}}^{\left(\max\right)} & =\max\left\{y_{\text{last}}^{t(\text{i})}\right\} \end{array} & \text{I}=1,\cdots N_{\text{dec}} \end{array}$$

where a superscript *t* indicates that the queue sorts the *N_dec_* LCDPs in time series. When the AUV approaches the odor source, the distances between the LCDPs become smaller, *i.e.*, the SIZ_T size becomes smaller. During CPT missions, the SIZ_T algorithm dynamically checks the diagonal:

(3) $$R=\sqrt{{\left(x_{\text{last}}^{\left(\text{max}\right)}-x_{\text{last}}^{\left(\text{min}\right)}\right)}^{2}+{\left(y_{\text{last}}^{\left(\text{max}\right)}-y_{\text{last}}^{(\min\text{)}}\right)}^{2}}$$

when *R*≤ ε*_T_*, SIZ_T identifies the mean:

(4) $$\begin{array}{cc} x_{\text{last}}^{\left(\text{M}\right)} & =\sum_{\text{I}=1}^{N_{\text{dec}}}x_{\text{last}}^{t(\text{i})}/N_{\text{dec}}\\ y_{\text{last}}^{\left(\text{M}\right)} & =\sum_{\text{I}=1}^{N_{\text{dec}}}y_{\text{last}}^{t(\text{i})}/N_{\text{dec}} \end{array}$$

as the source location. The SIZ_T algorithm needs three parameters: the criterion, *ε_T_*, for checking the SIZ_T size, the integer, *N_dec_*, indicating the constant number of LCDPs maintained in the queue, and the initial value, *N_ini_*, identifying the priority queue for accumulation of at least *N_ini_* LCPDs before starting source identification. The parameters *ε_T_* and *N_dec_* are adjustable and crucial to achieving the desired performance of source identification.

**Algorithm 1.** Pseudo Code of SIZ_T algorithm.
**ALGORITHM SIZ_T(***Q*[ 1,… *N_dec_* ] **)**
//Identifying the source location by **SIZ_T** algorithm//Input: Priority queue *Q*[ 1,… *N_dec_* ]//Output: Status of source identification **if** ( *N_dec_* ≥ *N_ini_* )  Sort *Q* in the order of the time serials  **for ***i* ← 1 **to ***N_dec_***do**   *P* [*i*] ← *Q*[*i* ] // *P* is a list   Calculate 
$\left(x_{\text{last}}^{\left(\min\right)},y_{\text{last}}^{\left(\min\right)}\right)$ and 
$\left(x_{\text{last}}^{\left(\max\right)},y_{\text{last}}^{\left(\max\right)}\right)$ in Equation (2)   Calculate the diameter of **SIZ_T** in Equation (3)   **if** the diameter ≤ *ε_T_*    **return**
$\left(x_{\text{last}}^{\left(\text{M}\right)},y_{\text{last}}^{\left(\text{M}\right)}\right)$ as the source location   **else**    **return** no source location identified **else**  **return** no source location identified

Algorithm 2 lists the pseudo code of the SIZ_F algorithm which maintains *all* LCDPs in the order of the current up-flow direction using the priority queue. SIZ_F holds a constant size, *ε_F_*, and makes the source identification by the following iterative construct: First, SIZ_F calculates $\left(x_{\text{last}}^{\left(\text{M}\right)},y_{\text{last}}^{\left(\text{M}\right)}\right)$ of all the LCDPs; Second, SIZ_F find the point, *p_max_*, with the largest distance to $\left(x_{\text{last}}^{\left(\text{M}\right)},y_{\text{last}}^{\left(\text{M}\right)}\right)$:

(5) $$D_{\text{max}}=\text{max}\left\{\sqrt{{\left(x_{\text{last}}^{f(\text{i})}-x_{\text{last}}^{\left(\text{M}\right)}\right)}^{2}+{\left(y_{\text{last}}^{f(\text{i})}-x_{\text{last}}^{\left(\text{M}\right)}\right)}^{2}}\right\}(\text{I}=1,2,\ldots N_{\text{all}})$$

from the priority queue, where a superscript *f* indicates that the LCDPs are sorted in the order of the most recent up-flow direction, and *N_all_* is the total number of LCDPs detected during a CPT mission. If *D_max_* is greater than, *ε_F_*, SIZ_F removes the LCDP with *p_max_* from the set of LCDPs. These calculations repeat until all remaining LCDPs are close enough to $\left(x_{\text{last}}^{\left(\text{M}\right)},y_{\text{last}}^{\left(\text{M}\right)}\right)$, *i.e.*, they all are located inside SIZ_F. If the number of the remaining LCDPs is greater than *N_min_*, SIZ_F identifies its most up-flow LCDP as the odor source. The SIZ_F algorithm has three parameters: the SIZ_F size, *ε_F_*, the initial value, *N_ini_*, and the integer, *N_min_*, which indicates the minimum number of LCDPs remaining inside SIZ_F for the source identification. The SIZ_F algorithm also has two the adjustable parameters *ε_F_* and *N_min_*. The parameter, *N_ini_*, defined in the algorithms works as a filter to block some invalid LCPDs only when *N_dec_* or *N_min_* is very small.

**Algorithm 2.** Pseudo Code of SIZ_F algorithm.
**ALGORITHM SIZ_F** ( *Q*[1,… *N_all_*] )
//Identifying the source location by **SIZ_F** algorithm//Input: Priority queue *Q*[1,… *N_all_*]//Output: Status of source identification **if** ( *N_all_* ≥ *N_ini_* )  Sort Q in the order of the current up-flow direction  *L*[1,… *N_all_*] ← *Q*[1,… *N_all_*]; *n*_1_ ← *N_all_* // *L* is a list  *status* ← **false**  **while ***n*_1_ ≥ *N_min_***do**   Calculate 
$\left(x_{\text{last}}^{\left(\text{M}\right)},y_{\text{last}}^{\left(\text{M}\right)}\right)$ of all **LCDP**s in the priority queue;   Find *p_max_* with *D_max_* in Equation (5)   **if ***D_max_* > *ε_F_*    remove *p_max_* from *L*; *n*_1_←*n*_1_-1   **else**    *status* ← **true**; **break**    **if ***status* = **true**     **return **
$\left(x_{\text{last}}^{f(1)},y_{\text{last}}^{f(1)}\right)$ as the source location   **else**    **return** no source location identified **else**  **return** no source location identified

We evaluate the SIZ algorithms using the simulated plume [23,24]. The simulated plume model achieves significant computational simplification relative to turbulence models, but it was designed to maintain the plume characteristics that significantly complicate the plume tracing problems (intermittency, meander, and varying flow) caused by natural flow fluid. Instead of adjusting the Reynolds numbers, it controls a filament release rate (5–10 filaments/s) to simulate filament intermittency and addresses the meandering nature of the plume as a key factor complicating the plume tracing. It also manipulates flow variation to challenge the CPT strategies. An operation area is specified by [0, 100] × [−50, 50] in meters. The simulation time step is 0.01 s and the mean fluid velocity is 1 m/s. A source location is chosen as (20, 0) in meters for checking the accuracy of the identified source locations, but it is unknown to the vehicle during CPT test runs. The simulation environment is set below: first, the filament release rate is 5 filaments/s because a low release rate may result in significant plume intermittency, which often causes the vehicle to lose contact with the plume.

The SIZ_T condition is stronger than the SIZ_F condition as SIZ_T requires the AUV to generate a few of the most recent LCDPs close enough in the vicinity of the odor source. However, it is not always true because the AUV may exit both sides of plumes in the vicinity of the source location due to the width of chemical plumes, the variation of flow directions, the intermittency of filaments, or the vehicle's mechanical restrains. For source declaration, SIZ_F needs a few of the most up-flow LCDPs close enough so it is suggested to use SIZ_F to identify a static plume source since the odor source located in the up-flow direction always makes the AUV progress up-flow.

## 4. In-Water Tests for Declaring Rhodamine Dye Plume Sources

**Figure 5.**
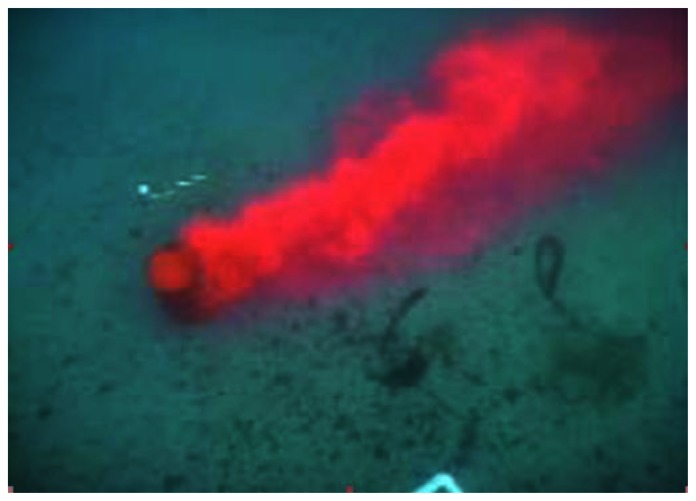
Rhodamine dye plume in the vincity of its source location (November 2002 at San Clemente Island, California, USA)

A type of SIZ_T algorithms is implemented on the REMUS vehicle for the CPT missions. The initial values of SIZ_T were chosen *ε_T_* =3 and *N_dec_* = 3 prior to the 2002 CPT missions. Eight of the first ten runs aborted due to vehicle issues, including start-up problems, incorrect time settings, equipment failure, *etc.* The other two test runs performed plume tracing well, but source declaration did not occur as both *ε_T_* and *N_dec_* were too small. We empirically changed to *ε_T_* =4 and *N_dec_* = 6 (our Monte Carlo evaluations in [25] show this set of parameters has a success rate of 96.8%). These settings successfully declared the source locations of rhodamine dye plumes, as shown in Figure 5, for the last seven test runs at the San Clemente Island (California, USA) in November 2002 labeled as MSN007r2–MSN010r3. Figure 1(a) shows the mission MSN007r2 that documented over a distance of 411 meters from the first detection point to the identified source location. In the in-water tests in April 2003 at San Clemente Island, the same settings successfully identified the source location on seven of eight experiments. The experiments included ground truth confirmation of identified source locations via sidescan sonar with 8–17 m accuracy. These settings were also successfully used for the in-water tests conducted in June 2003 in Duck (North Carolina, USA) in an operation area of 367 × 1,094 m (bigger than 60 football fields). Two types of missions were of interest during this set of experiments. The first mission type involved a single chemical source in the operation area. The second mission type may contain a few chemical sources in the operation area. The two types of CPT mission successfully identified the source locations with ground truth confirmation via sidescan sonar with 6–31 m accuracy [13]. Figure 1(b) shows the MSN003 mission at Duck in June 2003. This mission tracked the chemical plume for 976 m between the first detection point and the declared source location, with 13 m accuracy.

**Figure 6.**
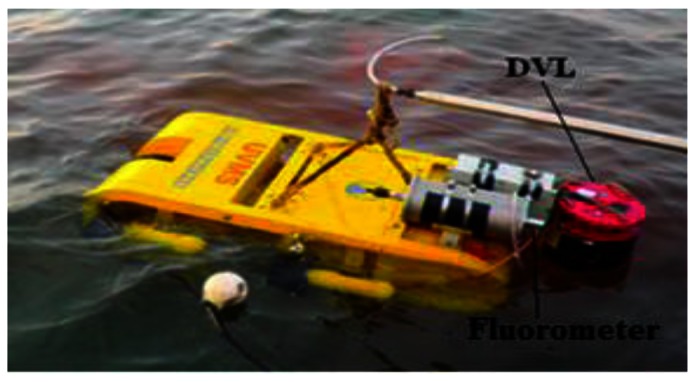
The AUV for the field experiments conducted in October 2010, developed by the Shenyang Institute of Automation, Chinese Academy of Sciences.

**Figure 7.**
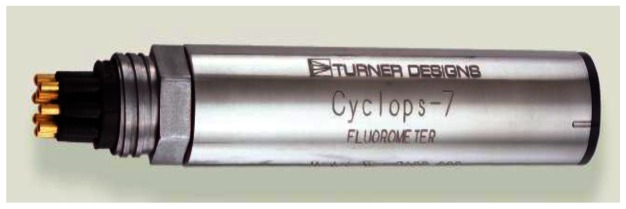
Cyclops-7 underwater fluorometer. For the in-water tests, the sampling rate of the fluorometer is set as 10 Hz, and the 0–10 μg/L measurement scale in which the fluorometer outputs 5 VDC corresponding to a rhodamine dye concentration of 10 μg/L is chosen.

**Figure 8.**
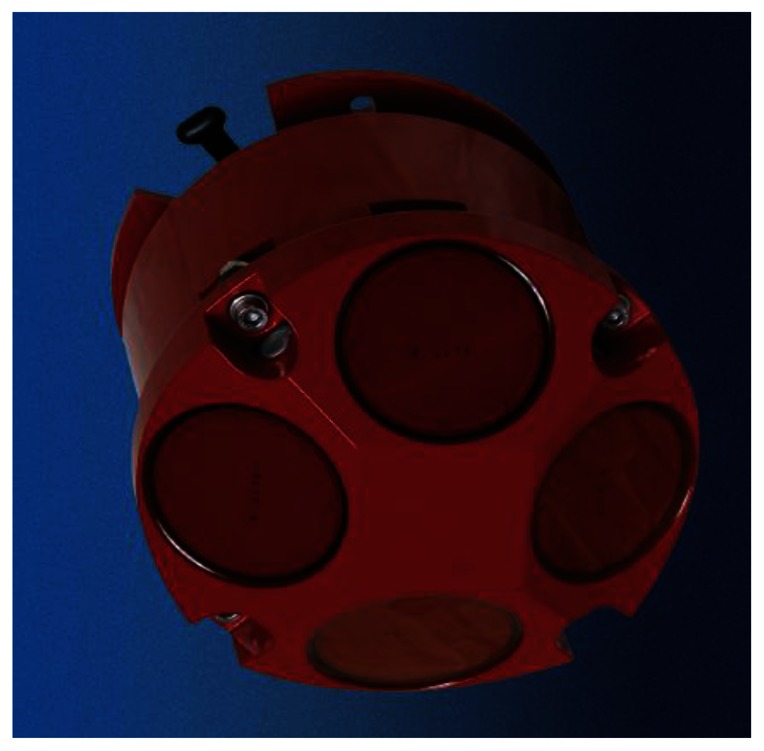
Workhorse navigator 1200K DVL.

The most recent in-water tests for identifying rhodamine dye plume sources were conducted in October 2010 at Dalian Bay (China). For this set of field tests, we implemented the SIZ_F algorithm on the AUV developed by SIA. The parameters *ε_F_* =6 and *N_min_*= 6 in SIZ_F are optimized in [12] prior to the 2012 CPT missions. The AUV shown in Figure 6 is equipped with multiple sensors, including a depth sensor, an underwater fluorometer, and a Doppler Velocity Log (DVL), *etc.* Figure 7 shows the fluorometer Cyclops-7 produced by Turner Designs, Inc. (Sunnyvale, CA, USA). Its technical specifications can be found at http://www.turnerdesigns.com/products/submersible/cyclops-7. The AUV uses the fluorometer to detect rhodamine dye plume concentration. For the in-water tests, we set the sampling rate of the fluorometer as 10 Hz and choose the 0–10 ug/L measurement scale in which the fluorometer outputs 5 VDC corresponding to a rhodamine dye concentration of 10 μg/L. Figure 8 shows the workhorse navigator 1200K DVL produced by Teledyne RD Instruments (Poway, CA, USA), and its technical specifications can be found at http://www.rdinstruments.com/pdfs/wh_navigator_ds_lr.pdf. The DVL measures the AUV speed relative to the sea bottom based on which we use the dead reckoning method to estimate AUV positions as well as fluid speed and orientation.

**Figure 9.**
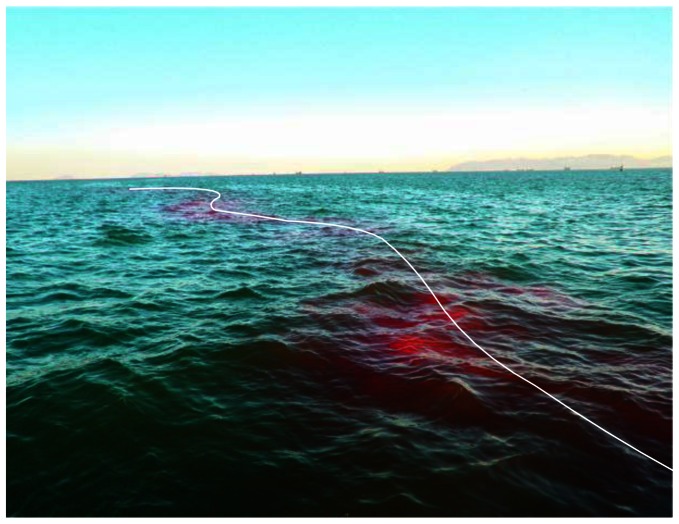
A rhodamine plume generated by a release rate of 1–2 g/min is observed about 350 meters far away from the source. This distance is calculated based on GPS signals.

For the in-water tests conducted in October 2010 at Dalian Bay (China), we developed an alternative strategy to generate rhodamine dye plumes on the sea surface, instead of on the seabed. The rhodamine dye is pumped up to the sea surface through a wound drainage plastic pipe. The pump was placed on a small boat that is anchored to the sea bottom. The plume source is 1.0 meters × 0.5 meters and the chemical source is submerged 1.5 meters below the sea surface. The rate of release of the rhodamine dye is about 1–2 g/min. Figure 9 shows a snapshot of the chemical plume taken at a location about 350 meters from its source. The scenario in Figure 9 provides the following information: first, a significant plume meander appears when the plume is propagated over a long distance. Changes in fluid flow direction cause the plume to meander (forming a snakelike path). Because the fluid flow direction and magnitude change, spatially and temporally, the instantaneous fluid flow direction within the plume often will not point toward the plume's source nor be coincident with the plume's centerline. To our knowledge, most of existing plume tracing algorithms do not explicitly discuss how to deal with the plume meander issue. Second, fluid flow waves affect the rhodamine dye plume so that the chemical concentration distribution is not uniform. The rhodamine dye plume in Figure 8 shows the chemical concentration at some locations where the plume looks like it is broken and the color is very low, but at other locations with a large dye area the level of deep red color is very high. The problem with local concentration maxima significantly challenges source declaration algorithms which are investigated in laboratory environments by searching for the maximum proximity of a plume with a uniform concentration distribution. For identifying the source locations, the AUV started tracing the rhodamine dye plumes developed on the sea surface. In this case, we visually observe the AUV maneuvers and confirm the identified chemical source locations via a GPS. Two among the six test runs aborted due to equipment failure instead of the SIZ_F settings. The mission, labeled as MSN171639, successfully tracked the chemical plume to its source location and identified the source locations with accuracy 8.38 m relative to the source locations confirmed by the GPS, as shown in Figure 2. The declared source location accuracies of the missions, labeled as MS10171617, MS10191502, and MS10191536, are 15.77 m, 12.33 m, and 28.42 m, respectively. The work in [12] developed a time metric to estimate the identification time cost (*N_min_*−1)*κ which is free from plume mission initial positions. κ is about 94.5 s, determined by the vehicle velocity and turn radius. The identification time costs’ average is close to the estimated one. The source declaration algorithms allow little room for the improvement of time cost if only a single chemical sensor is used.

Neutral buoyancy of the chemical or stratification of the flow will often result in the rhodamine dye plume of limited vertical extent, which may be approximated as a two dimensional (2-D) problem. In the field tests, no test runs failed due to the 3D problem. For searching for hydrothermal vents in oceans, the 3D issue has to be considered.

## 5. Source Verification Module

For further operations to deal with the declared plume source, the source needs to be automatically or interactively confirmed or inspected using a visual system. Images taken in near shore ocean environments are very vague and colors in the images are not well-defined since the propagation of light underwater is affected by scattering, refraction, and absorption. However, most of the existing techniques [26–28] provide crisp segmentation of images, where each pixel is classified into a unique subset. This classification may not be effective for extracting a rhodamine dye plume and its source in underwater conditions because colors in the images taken in near shore ocean environments are not well-defined.

In order to deal with uncertainty, we use a fuzzy logic based iterative algorithm to segment color components of the rhodamine dye plume and its source [29]. In this study, colors of an image are described in the RGB space. The color of each pixel p(*m*, *n*) denoted by p(*m*, *n*)_RGB_ is processed to separate its red, green, and blue components {p(*m*, *n*)_R_, p(*m*, *n*)_G_, p(*m*, *n*)_B_}. The fuzzy color extractor extracts a cluster of colors based on a defined color pattern (CP or CP_RGB_). The CP_RGB_ is either directly defined by its RGB components (CP_R_, CP_G_, CP_B_) or determined by a pixel in the image. The color component differences between p(*m*, *n*)_RGB_ and CP_RGB_ are calculated as follows:

(6) $$\{\begin{array}{l} \text{dif}{\left(\text{M},n\right)}_{\text{R}}=p{\left(\text{M},n\right)}_{\text{R}}-{\text{CP}}_{\text{R}}\\ \text{dif}{\left(\text{M},n\right)}_{G}=p{\left(\text{M},n\right)}_{G}-{\text{CP}}_{G},0\leq \text{M}<\text{M},0\leq n<N,\\ \text{dif}{\left(\text{M},n\right)}_{B}=p{\left(\text{M},n\right)}_{B}-{\text{CP}}_{B} \end{array}$$

where *M* and *N* indicate the size of an array which holds the image. The following fuzzy rules are applied to dif (*m, n*)_R_, dif (*m, n*)_G_, and dif (*m, n*)_B_:
*If**dif* (*m*, *n*)_R _*and dif* (*m*, *n*)_G _*and dif* (*m*, *n*)_B _*are* Zero*Then*p(*m*, *n*) *is* Matched*If**dif* (*m*, *n*)_R _*or dif* (*m*, *n*)_G _*or dif*(*m*, *n*)_B _*is* Negative *or* Positive*Then*p(*m*, *n*) *is* Unmatched

**Figure 10.**
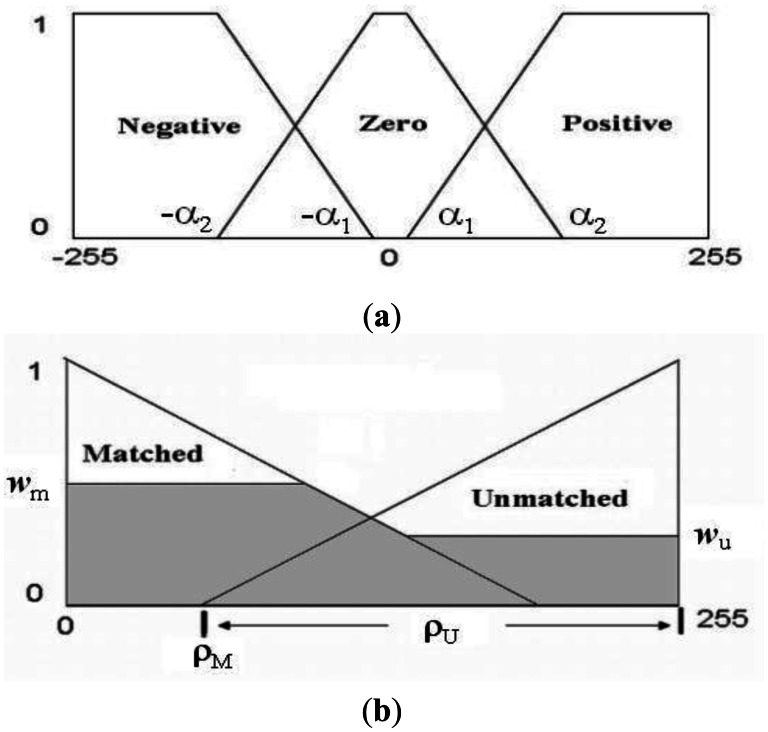
Membership functions for color image segmentation. (**a**) Membership functions for color differences; (**b**) Membership functions for defuzzification.

Both rules indicate that the pixel, p(*m*, *n*), belongs to the object to be extracted, if the Euclidean distances between p(*m*, *n*)_RGB_ and CP_RGB_ along the three axes in RGB coordinate system are small enough; otherwise, p(*m*, *n*) does not belong to the object. Figure 10(a) shows the membership functions (μ_N_(*x*), μ_Z_(*x*), μ_P_(*x*)) for the input fuzzy variables (Negative, Zero, Positive) defined by:

(7) $$\mu_{\text{N}}(x)=\{\begin{array}{l} 1-255\leq x<-\alpha_{2}\\ \frac{\left(x+\alpha_{1}\right)}{\left(\alpha_{1}-\alpha_{2}\right)}-\alpha_{2}\leq x<-\alpha_{1}\\ 0-\alpha_{1}\leq x\leq -255 \end{array}$$

(8) $$\mu_{\text{Z}}(x)=\{\begin{array}{l} 0-255\leq x<-\alpha_{2}\\ \frac{\left(x+\alpha_{2}\right)}{\left(\alpha_{2}-\alpha_{1}\right)}-\alpha_{2}\leq x<-\alpha_{1}\\ 1-\alpha_{1}\leq x<-\alpha_{1}\\ \frac{\left(x+\alpha_{1}\right)}{\left(\alpha_{2}-\alpha_{1}\right)}\alpha_{1}\leq x<-\alpha_{2} \end{array}$$

(9) $$\mu_{\text{p}}(x)=\{\begin{array}{l} 0-255\leq x<\alpha_{1}\\ \frac{\left(x+\alpha_{1}\right)}{\left(\alpha_{2}-\alpha_{1}\right)}\alpha_{1}\leq x<\alpha_{2}\\ 1\;\alpha_{2}\leq x\leq 255 \end{array}$$

Figure 10(b) shows the membership functions (μ_M_(*x*), μ_U_(*x*)) for the output fuzzy variables (Matched, Unmatched) defined by:

(10) $$\mu_{\text{M}}(x)=\{\begin{array}{l} \frac{\left(\rho_{\text{M}}-x\right)}{\rho_{\text{M}}}0\leq x<\rho_{\text{M}}\\ 0\;\rho_{\text{M}}\leq x\leq 255 \end{array}$$

(11) $$\mu_{\text{U}}(x)=\{\begin{array}{l} 0\;0\leq x<\rho_{\text{U}}\\ \frac{\left(x-\rho_{\text{U}}\right)}{\left(255-\rho_{\text{U}}\right)}\rho_{\text{U}}\leq x\leq 255 \end{array}$$

where ρ_M_ + ρ_U_ = 255. Based on *dif* (*m*, *n*)_R_, *dif* (*m*, *n*)_G_, and *dif* (*m*, *n*)_B_, the fuzzy rules produce the weight *w*_m_ for Matched and the weight *w*_u_ for Unmatched by:

(12) $$\begin{array}{c} w_{\text{M}}=\text{min}\{w_{\text{M}(\text{R})},w_{\text{M}(\text{G})},w_{\text{M}(\text{B})}\}\\ w_{\text{U}}=\text{max}\{w_{\text{U}(\text{R})},w_{\text{U}(\text{G})},w_{\text{U}(\text{B})}\} \end{array}$$

Figure 10(b) also shows the produced areas in the output domain while *w*_m_ and *w*_u_ cutting μ_M_(*x*) and μ_U_(*x*). An output value, Δρ_F_, is calculated by the centroid defuzzification method:

(13) $$\Delta \rho_{F}=\frac{\int \mu_{\text{out}}(x)xdx}{\int \mu_{\text{out}}(x)dx}$$

where μ_out_ (*x*) represents the envelope function of the areas cut by *w*_m_ and *w*_u_ in fuzzy output domain. If Δρ_F_ < δ, p(*m*, *n*) is extracted; otherwise, p(*m*, *n*) is not extracted, where δ is a threshold. The fuzzy color extractor can be understood as a mapping operator between Euclidean distances {*dif*(*m*, *n*)_R_, *dif*(*m*, *n*)_G_, *dif*(*m*, *n*)_B_} in the RGB space and a difference Δρ_F_ in the intensity space under a fuzzy metric. We use the procedure **FuzzyColorSeg** to the extract specified color from a color image.

**FuzzyColorSeg**( I_S_ ) CP_RGB_ ← **GetCP**( I_S_ );  [I_U_, I_M_] ← **FCE**( I_S_, CP_RGB_ ); **return** [I_U_, I_M_];

**FuzzyColorSeg** invokes two procedures: **GetCP** and **FCE**. **GetCP** generates a CP_RGB_ for **FCE** which splits a source image I_S_ into two sub-images: one sub-image, I_M_, holds the matched colors; and the other sub-image, I_U_, remains the unmatched colors.

**Figure 11.**
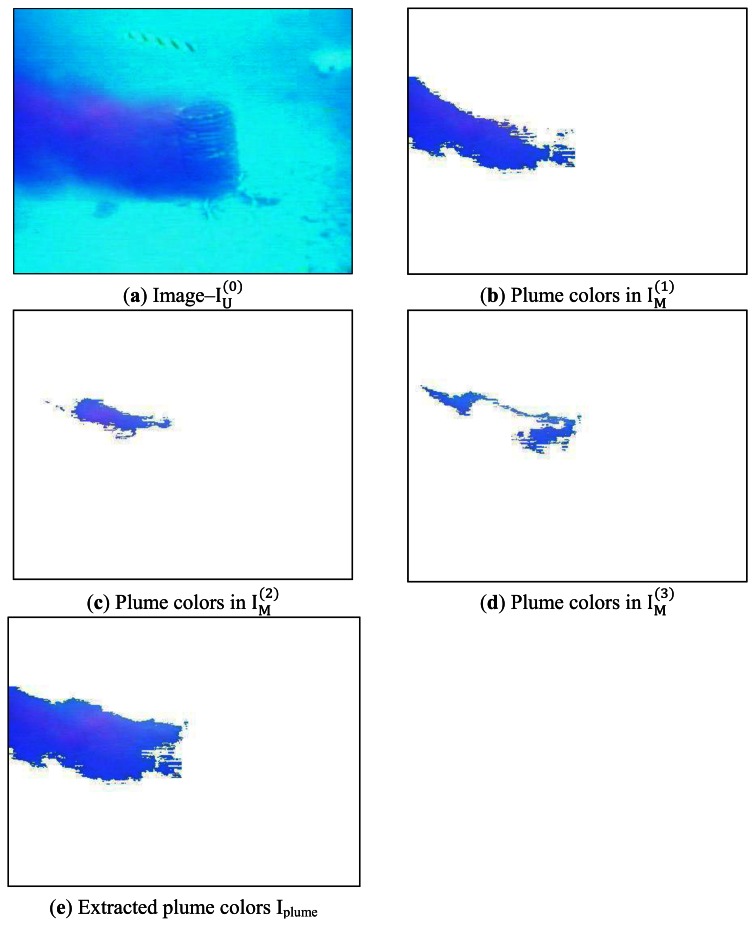

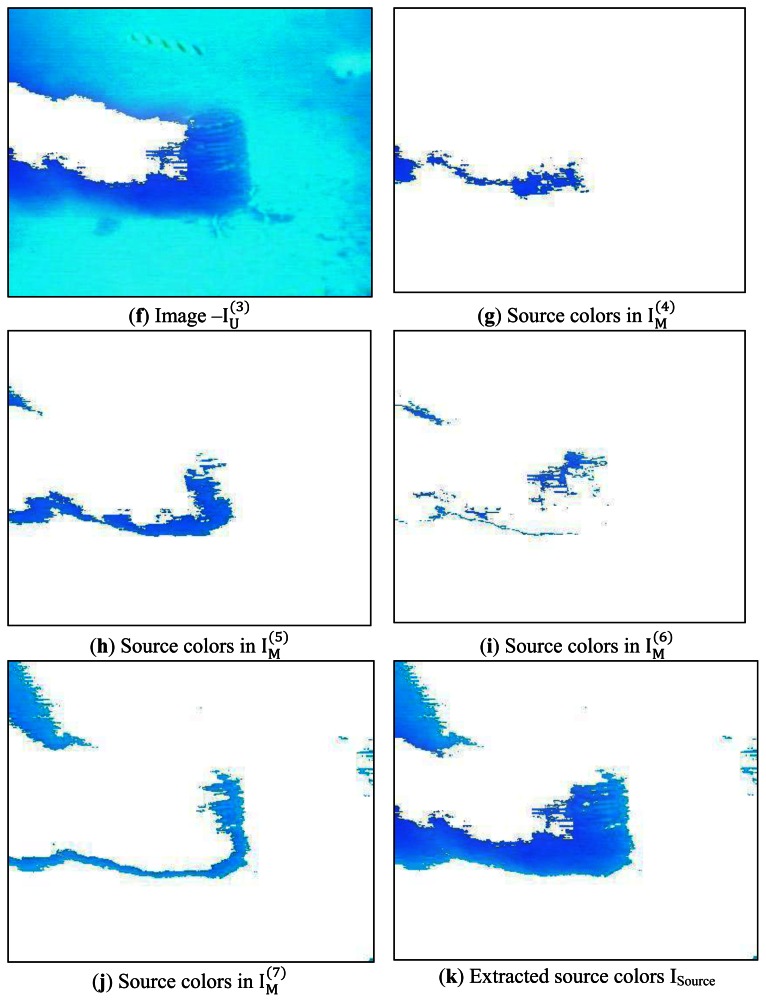
Extraction of the rhodamine dye plume and its source.

The difficulty of segmenting the chemical plume and its source from images taken in ocean environments lies in the definition of their color patterns, since colors in the vague images are not well defined due to illumination variance and fluid advection affects. Now, we discuss an iterative procedure to extract the rhodamine dye plume and its source from such a color image taken in near-shore oceanic environments. For convenience, we define two groups of the sub-images ${\text{I}}_{\text{M}}^{\left(\text{i}\right)}$ and ${\text{I}}_{\text{U}}^{\left(\text{i}\right)}$ split by **FCE**. ${\text{I}}_{\text{M}}^{\left(\text{i}\right)}$ contains matched colors after the *i*th step extraction, while ${\text{I}}_{\text{U}}^{\left(\text{i}\right)}$ contains unmatched colors. The original image ${\text{I}}_{\text{U}}^{\left(0\right)}$ is the initial source image to the procedure, and ${\text{I}}_{\text{U}}^{\left(\text{i}\right)}$ is the source image for the (*i* + 1)th step segmentation. We have the relationship ${\text{I}}_{\text{U}}^{\left(\text{i}\right)}={\text{I}}_{\text{U}}^{\left(\text{i}+1\right)}\cup {\text{I}}_{\text{M}}^{\left(\text{i}+1\right)}$. In our application, a default color is defined as white with (255, 255, 255) in the RGB space. **FCE** generates ${\text{I}}_{\text{M}}^{\left(\text{i}\right)}$ by moving the matched pixels from the source image to an image initialized by white color, and generates ${\text{I}}_{\text{U}}^{\left(\text{i}\right)}$ by setting the extracted pixels in the source image to white color. Obviously, selecting color patterns becomes the key issue of extracting desired objects from an image. We employ some well-defined colors as reference colors to generate CPs. To our knowledge, the color components of the rhodamine dye plume are close to the red color, and those of the chemical source are dark and close to the blue color. Therefore, we define the two reference colors: RED_RGB_ with (255, 0, 0) and DARK-BLUE_RGB_ with (BLACK_RGB_+BLUE_RGB_)/2 = (0, 0, 128) to generate color patterns by using GetCP( I_s_ ):
RefColor ← RED_RGB_ or DARK-BLUE_RGB_; ‖CP_RGB_‖ ← ∞;**for ***m* ← 0 to *M*-1 do**for ***n* ← 0 to *N*-1 do Dis ← ‖RefColor - p(*m*, *n*)_RGB_‖ **if** Dis < ‖CP_RGB_‖CP_RGB_ ← p(*m*, *n*)_RGB_;**return** CP_RGB_;where I_s_ is an original image to be processed, *M* and *N* determine the size of I_s_. In order to extract color components of the chemical plume, RED_RGB_ is assigned to RefColor, and the procedure returns a CP_RGB_ that holds the color components of the pixel in the image with the shortest Euclidean distance to the reference color – RED_RGB_. ${\text{I}}_{\text{U}}^{\left(0\right)}$ is the original image–“odor”, as shown in Figure 11(a), and ${\text{I}}_{\text{M}}^{\left(1\right)}$ is initialized as an empty image with white. **FuzzyColorSeg** takes ${\text{I}}_{\text{U}}^{\left(0\right)}$ as an input and passes ${\text{I}}_{\text{U}}^{\left(0\right)}$ to **GetCP**. **GetCP** defines RED_RGB_ as RefColor and returns a CP_RGB_ (106, 106, 231) for ${\text{I}}_{\text{U}}^{\left(0\right)}$. **FCE** uses the CP_RGB_ to split ${\text{I}}_{\text{U}}^{\left(0\right)}$ –“odor” into two sub-images ${\text{I}}_{\text{U}}^{\left(1\right)}$ and ${\text{I}}_{\text{M}}^{\left(1\right)}$. ${\text{I}}_{\text{M}}^{\left(1\right)}$ is produced by moving the extracted pixels from ${\text{I}}_{\text{U}}^{\left(0\right)}$ to the empty image and holds color components closely matching to the CP_RGB_. ${\text{I}}_{\text{U}}^{\left(1\right)}={\text{I}}_{\text{U}}^{\left(0\right)}-{\text{I}}_{\text{M}}^{\left(1\right)}$ is produced by setting the extracted pixels to white in ${\text{I}}_{\text{U}}^{\left(0\right)}$. ${\text{I}}_{\text{M}}^{\left(1\right)}$ is the first segmented image for the chemical plume, as shown in Figure 11(b). The chemical plume in the image is not completely extracted since the rhodamine dye colors are distorted due to environmental illumination variation and fluid advection affects. The further segmentation needs ${\text{I}}_{\text{U}}^{\left(1\right)}$ as a new source image for the next step segmentation. **FuzzyColorSeg** takes ${\text{I}}_{\text{U}}^{\left(1\right)}$ as its input, and **GetCP** gets a new CP_RGB_ with (156, 136, 243) for ${\text{I}}_{\text{U}}^{\left(1\right)}$. **FCE** uses the updated CP_RGB_ to split the image ${\text{I}}_{\text{U}}^{\left(1\right)}$ into two sub-images ${\text{I}}_{\text{U}}^{\left(2\right)}$ and ${\text{I}}_{\text{M}}^{\left(2\right)}$. Similarly, ${\text{I}}_{\text{M}}^{\left(2\right)}$ is produced by moving the extracted pixels from ${\text{I}}_{\text{U}}^{\left(1\right)}$ to an empty image and holds the color components closely matching to the updated CP_RGB_. ${\text{I}}_{\text{M}}^{\left(2\right)}$ represents the second segmented image for the chemical plume, as shown in Figure 11(c). ${\text{I}}_{\text{U}}^{\left(2\right)}$ is generated by setting the extracted pixels from ${\text{I}}_{\text{U}}^{\left(1\right)}$ to white. At the *i*th step, **FuzzyColorSeg** split ${\text{I}}_{\text{U}}^{\left(\text{i}\right)}$ into ${\text{I}}_{\text{U}}^{\left(\text{i}+1\right)}$ and ${\text{I}}_{\text{M}}^{\left(\text{i}+1\right)}$. Figure 11(d) displays the segmented image ${\text{I}}_{\text{M}}^{\left(3\right)}$ at the third step. The union operation ${\text{I}}_{\text{plume}}={\text{I}}_{\text{M}}^{\left(1\right)}\cup {\text{I}}_{\text{M}}^{\left(2\right)}\cup {\text{I}}_{\text{M}}^{\left(3\right)}$ generates the segmented chemical plume, as shown in Figure 11(e).

Similarly, we use **FuzzyColorSeg** to extract the color components of the chemical source from the image. In this case, DARK-BLUE_RGB_ is assigned to **RefColor** to generate a CP_RGB_. **FCE** uses this CP_RGB_ split ${\text{I}}_{\text{U}}^{\left(3\right)}$ into two sub-images ${\text{I}}_{\text{U}}^{\left(4\right)}$ and ${\text{I}}_{\text{M}}^{\left(4\right)}$, as shown in Figure 11(f). This procedure continues four steps to extract the color components of the chemical source, as shown in Figure 11(f–j). The union operation ${\text{I}}_{\text{source}}={\text{I}}_{\text{M}}^{\left(4\right)}\cup {\text{I}}_{\text{M}}^{\left(5\right)}\cup {\text{I}}_{\text{M}}^{\left(6\right)}\cup {\text{I}}_{\text{M}}^{\left(7\right)}$ generates the extracted color components of the chemical source shown in Figure 11(k).

The extracted colors in ${\text{I}}_{\text{M}}^{\left(\text{i}\right)}$ can be understood as a fuzzy color cluster under the given membership function. For segmenting colors in the images in Figure 11, we choose the parameters of the membership functions as follows: α_1_ = 35, α_2_ = 200, ρ_M_ = 20, ρ_U_ = 235, and ɛ = 80. In this study, we consider two criteria for clustering the colors extracted by **FCE** [30]: The first is based on the distances of the CPs to their associated reference color, and the second based on the relative distances between the CPs. For the first criterion, we take the CP_RGB_ with the shortest distance to the given reference color as a pivot. Then, we calculate the differences between distances of the pivot and any other CPs in this group: Δ_1_ = ‖CP_RGB_‖−‖Pivot_RGB_‖. If Δ_1_ > δ_1_, its corresponding CP_RGB_ is excluded from this group. For the second criterion, we construct a graph based on the distances between the CPs in terms of the given reference color. Then, we start with the pivot in the graph to calculate the shortest distances using Floyd's algorithms. The CP_RGB_ is removed from this graph, if its shortest distance is greater than δ_2_. Both the criteria ensure that the CPs in each group are close to their pivot enough. In this study, we select δ_1_ and δ_2_ as 40 and 45.

## 6. Conclusions

This article discusses a strategy for identifying a chemical source in a near-shore and ocean environment by integration of chemical and visual sensors. The two source identification algorithms SIZ_T and SIZ_F are abstracted from the moth-inspired plume tracing strategies presented in [2]. While animal plume tracing relies primarily on sensed pheromones, the final determination of the plume source location could be based on data from multiple sensors, including vision, tactile or even auditory cues [22]. Typically, olfactory-based mechanisms proposed for biological entities combine a large-scale orientation behavior based in part on olfaction with a multisensor local search in the vicinity of the source.

Extracting the chemical plume and its source from an image taken in underwater conditions is difficult since the color images taken in near-shore oceanic environments are very vague and the objects’ colors in the image are significantly distorted from their natural colors due to dim illumination conditions and flow fluid influence. In order to deal with uncertainty, segmenting color components of the chemical plume and its source is based on the fuzzy color extractor. The fuzzy color extractor is directly extended from the fuzzy gray-level extractor, which was applied to recognize while line landmarks on roads for autonomous vehicle navigation [31,32]. The verification module was used to test five images captured from the video made during the in-water of 2002 and accomplished the plume source extraction. Evaluations of the module in different environment conditions are planned to test its robustness and effectiveness further. The autonomous underwater vehicle for our ongoing project on searching for hydrothermal vents in oceans will carry an underwater camera and a lightning system to get images during operation. An alternative sensor, e.g., sidescan sonar, can be used to confirm the declared source location if an image of the source is unavailable during CPT missions.

For the in-water tests, the CPT algorithm has to be able cope with significant plume meander. The simulation studies show that the moth-inspired CPT strategy is effective for tracing the plume with significant meander so we selected it for implementation. By considering time-consumption and cost of the field tests, no other algorithms are selected for the in-water tests.

The source identification performance can be improved if the visual sensor based source identification model checks each LDCP. This visual sensor based system has a number of potential applications in our on-going project, e.g., it will guide a manipulator to get samples at thermal vents. We will investigate the fuzzy color segmentation algorithm by considering location and texture information. In our further research, we will also improve the source declaration performance by adaptively calculating the parameters of SIZ algorithms and consider the algorithms for tracking of the plume in 3D environment.
